# Effects of Expressive Writing on Neural Processing During Learning

**DOI:** 10.3389/fnhum.2019.00389

**Published:** 2019-11-06

**Authors:** Brynne C. DiMenichi, Ahmet O. Ceceli, Jamil P. Bhanji, Elizabeth Tricomi

**Affiliations:** Department of Psychology, Rutgers University, Newark, NJ, United States

**Keywords:** expressive writing, fMRI, learning, feedback, striatum, MCC, medial cingulate cortex

## Abstract

Expressive writing about past negative events has been shown to lead to a slew of positive outcomes. However, little is known about why writing about something negative would have positive effects. While some have posited that telling a narrative of a past negative event or current anxiety “frees up” cognitive resources, allowing individuals to focus more on the task at hand, there is little neural evidence suggesting that expressive writing has an effect on cognitive load. Moreover, little is known about how individual differences in the content of expressive writing could affect neural processing and the cognitive benefits writing confers. In our experiment, we compared brain activity in a group that had engaged in expressive writing vs. a control group, during performance on a feedback-based paired-associate word-learning task. We found that across groups, differential activation in the dorsal striatum in response to positive vs. negative feedback significantly predicted better later memory. Moreover, writing about a past failure resulted in more activation relative to the control group during the learning task in the mid-cingulate cortex (MCC), an area of the brain crucial to processing negative emotion. While our results do not provide support for the assertion that expressive writing alters attentional processing, our findings suggest that choosing to write about particularly intense past negative experiences like a difficult past failure may have resulted in changes in neural activation during task processing.

## Introduction

Expressive writing about a past negative experience has been shown to lead to a slew of positive outcomes. For example, writing about a past trauma has led to reductions in anxiety and depression (Lepore and Smyth, 2002; Smyth et al., 2008), as well as improvements in physical health (Pennebaker et al., 1988; Harber and Pennebaker, 1992). Writing about anxieties has also resulted in improved cognitive performance, both in the laboratory (Klein and Boals, 2001; DiMenichi and Richmond, 2015; DiMenichi et al., 2018) as well as in the classroom on high stakes exams (Ramirez and Beilock, 2011).

What is it about writing about negative experiences that leads to such benefits? While there is some evidence that writing about a past negative event leads to reductions in the physiological stress response (DiMenichi et al., 2018), some have posited that writing down negative feelings “frees up” cognitive load to better focus on the task at hand (Klein and Boals, 2001; Ramirez and Beilock, 2011), thus leading to observed performance benefits. However, there is little empirical evidence regarding how expressive writing relates to cognitive processing in the brain, as well as other brain networks vital to cognition. If writing about negative experiences like past traumas or current anxieties “frees up” cognitive resources, one would subsequently expect to see less activation in areas of the brain typically correlated with cognitive load, such as the dorsolateral prefrontal cortex (dlPFC; Rypma et al., 2002). Yet, no expressive writing intervention initiatives have examined how expressive writing affects neural processing, so it remains difficult to draw firm conclusions that changes in cognitive load are the mechanism behind the success brought about by expressive writing.

Alternatively, writing about a negative event like a past failure might lead to performance improvements *via* changes to affective or emotional processes. It is important to note that simply inducing a sad mood through writing has not been shown to improve cognitive performance, whereas writing about a negative event specific to the self, such as a failure, has been shown to confer cognitive benefits (DiMenichi et al., 2018). It is possible that being reminded of a past negative experience may cause an individual to be more sensitive to a new negative experience, such as negative feedback about performance. Therefore, we might expect to see changes in activation in the striatum, an area of the brain primarily associated with processing affective information, such as monetary rewards and punishments (Delgado et al., 2000, 2003), and positive and negative feedback about performance during learning (Tricomi and Fiez, 2012; DePasque and Tricomi, 2015; Lempert and Tricomi, 2016).

Furthermore, expressive writing about negative events or current worries may evoke strong emotions, which may alter neural activation in areas of the brain that typically process strong negative emotion. Thus, we hypothesized that changes in affective or emotional processing may be responsible for the benefits of expressive writing. However, without empirical evidence from the brain, it is difficult to draw conclusions about this proposed mechanism behind the success of expressive writing.

### Individual Differences in Brain Processing

While differences in brain processing between writing groups may help us gain insight as to the benefits of expressive writing about past failures, individual differences in the quality, stressfulness, intensity, or other aspects of their expressive writing sample may help us understand what specifically about writing about past failure guides performance improvements. Considering that writing about more intense failures has previously led to greater health benefits (Harber and Pennebaker, 1992; Pennebaker, 1997), one would expect that writing about a more stressful or intense failure may also result in greater immediate benefits to cognitive performance. Furthermore, information about individual differences in brain processing, and how these differences in brain processing relate to performance, may help us gain further information about the mechanism behind the benefits of expressive writings about past failures.

### Current Study

In this experiment, we examined how writing about a past failure affected both cognitive performance and neural processing on a feedback-based paired-association word-learning task. In previous studies using this task, distinct neural signatures in the striatum to positive vs. negative performance feedback have been elicited, in addition to engagement of a host of brain regions typically associated with the cognitive processes underlying effortful encoding (Tricomi and Fiez, 2012; DePasque and Tricomi, 2015; Lempert and Tricomi, 2016). We hypothesized that writing about a difficult time in which one did not succeed would result in better memory both during the word-learning task, as well as at a later surprise recall task, with changes in brain activation predicting these group performance differences. In particular, we hypothesized that there might be group differences in the neural responses to positive and negative feedback, or in activation of brain regions underlying effortful cognition. Alternatively, we predicted that individual differences in the quality of writing samples about past failures could predict individual differences in neural processing, which could, in turn, predict subsequent memory differences on our task.

## Materials and Methods

### Participants

Forty right-handed adults (24 female, 16 male) aged 18–35 were recruited from the surrounding area of Rutgers University-Newark. Sample size was determined based on a behavioral pilot study examining the effect of our writing manipulation on test performance in our task (see Supplementary Material). The pilot produced an effect size of *d* = 0.93. At 80% power and with an alpha of 0.05, this suggests a sample size of 40 (20 per group), which is the recommendation we used for our experiment. Participants (mean age = 22.23, SD = 3.81) reported to the Rutgers University Brain Imaging Center (RUBIC, Newark, NJ, USA). Participants were paid $50 for their participation. All participants gave written informed consent. The experiment was approved by the institutional review board of Rutgers University.

### Writing Task

Before the start of the scan, participants completed a writing manipulation adapted from DiMenichi and Richmond (2015). In the “failure” condition, participants saw a prompt on a computer screen that asked them to spend the next 10 min writing about a difficult time in which they did not succeed. They typed their response in the computer. Participants pseudo-randomly assigned to the “control” condition were prompted to write about the plot of a movie they had recently viewed. The goal of the control condition was to control for the general effects of writing on performance.

### Paired-Association Word Learning Task

**Figure 1:**
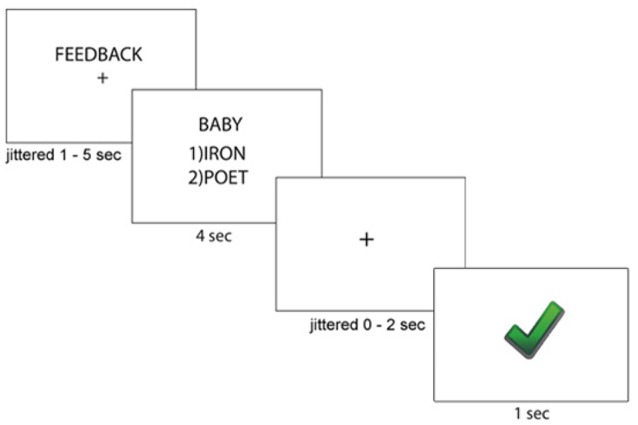
Paired-Association Word Learning Task Description. Participants were told whether they would receive feedback for the current block. Then, participants viewed a target word with two word choices, and pressed the key responding to their word choice. Participants then saw immediate accurate feedback regarding their word choice.

**Figure 2:**
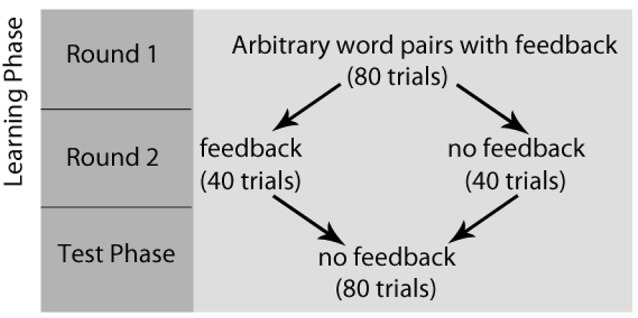
Paired-Association Word Learning Task Experimental Design. Participants completed two rounds of the learning phase, followed by the test phase. In the first round of the learning phase, word matches were arbitrary, but participants received feedback for all trials and were told to use this feedback for future rounds. After completing both rounds of the learning phase, participants completed the “test phase,” which asked participants to select the word that matched the target (without receiving any feedback) and rate their confidence in their response.

After completing the writing task, all participants completed a paired-association learning task with and without feedback inside the magnetic resonance imaging (MRI) scanner (Tricomi and Fiez, 2012; Lempert and Tricomi, 2016). In the “learning phase” of the experiment, participants viewed a “target” word with two arbitrary word choices below each target, and participants were told to select the word that matched the target word. Before each set of trials, participants were shown a label indicating if the block contained “feedback” or “no feedback.” In the feedback block, participants were given accurate feedback about their response—a green check mark if they were correct, or a red “X” if they were incorrect. In the no-feedback condition, participants saw a pound sign after their response; see Figure 1 for task description. Participants completed two rounds during the learning phase of the experiment. In the first round of the learning phase, word matches were new (and therefore arbitrary), but participants received feedback for all trials and were told to use this feedback for future rounds. Trials for which participants did not respond within the 4 s window were repeated at the end of each round. After completing both rounds of the learning phase with the same set of words, participants completed the “test phase” on the words from the task outside the scanner, which asked participants to select the word that matched the target(without receiving any feedback) and rate their confidence in their response on a Likert scale (1 = complete guess, 7 = completely sure); see Figure 2 for experimental design.

### Survey Battery

After the conclusion of the surprise recall task, participants provided demographic information, as well as several surveys corresponding to traits that could possibly affect our writing manipulation. The battery included the Connor-Davidson Resilience Scale, which measures individual differences in trait resiliency (Connor and Davidson, 2003); the Achievement Goal Questionnaire, which examines preference for wanting to achieve goals in order to master a new skill, perform well, or avoid failure (Elliot and Church, 1997); and the Need For Cognition Scale (NFC), which measures the tendency for an individual to prefer to engage in thinking (Olson et al., 1984). We also included the Cognitive Inference Questionnaire (CIQ), which asks participants to indicate how often they had thoughts that could have interfered with performance—e.g., worries about task performance, thoughts about things other than the task, etc. (Sarason et al., 1986). The Marlow-Crowne Social Desirability Scale was also included to measure any bias in responding to the survey battery (Crowne and Marlowe, 1960). Surveys were completed on a computer *via* the website Qualtrics (Provo, Utah) and presentation order was randomized by the computer to prevent order effects.

Lastly, we probed participants about their subjective experience of different aspects of the task. Specifically, we asked participants to rate how much they enjoyed and cared about doing well on the word-learning task, if they preferred negative to no feedback on the task, and to rate their level of stress upon arrival, during the writing task, while completing the writing task, and while completing the survey. We also asked participants how stressed they felt when the original event they wrote about occurred (either their past failure or movie). Specifically, participants were asked, “Please rate how stressful the event was WHEN IT ORIGINALLY OCCURRED.”

### fMRI Data Collection and Analyses

We utilized a 3 Tesla Siemens TRIO scanner and 12 channel head coil at the RUBIC. Stimulus presentation was implemented with E-Prime Experimental Software (Psychology Software Tools, Pittsburgh, PA, USA), and functional magnetic resonance imaging (fMRI) data were preprocessed and analyzed using BrainVoyager QX 2.3.1 Software (Brain Innovation, Maastricht, The Netherlands). Anatomical slices were collected using a T1-weighted protocol of 176 isotropic 1-mm voxel sagittal slices, while functional slices were collected using a single-shot EPI pulse sequence with a TR of 2,500 ms and TE of 25 ms. Forty-one contiguous oblique-axial 3 mm × 3 mm × 3 mm voxel slices were acquired in an oblique orientation of 30° to the anterior commissure-posterior commissure (AC-PC) axis. This orientation has been found to reduce signal dropout in the ventral prefrontal cortex (vPFC; Deichmann et al., 2003).

During analysis, fMRI data were normalized to the Talairach stereotaxic space (Talaraich and Tournoux, 1988) before preprocessing. Preprocessing included slice-time correction, motion correction, 4 mm spatial smoothing, and high-pass temporal filtering (high pass GLM-Fourier, 3 sines/cosines, 3 s). Preprocessed data was then analyzed using a random-effects general linear model (GLM).

**Figure 3:**
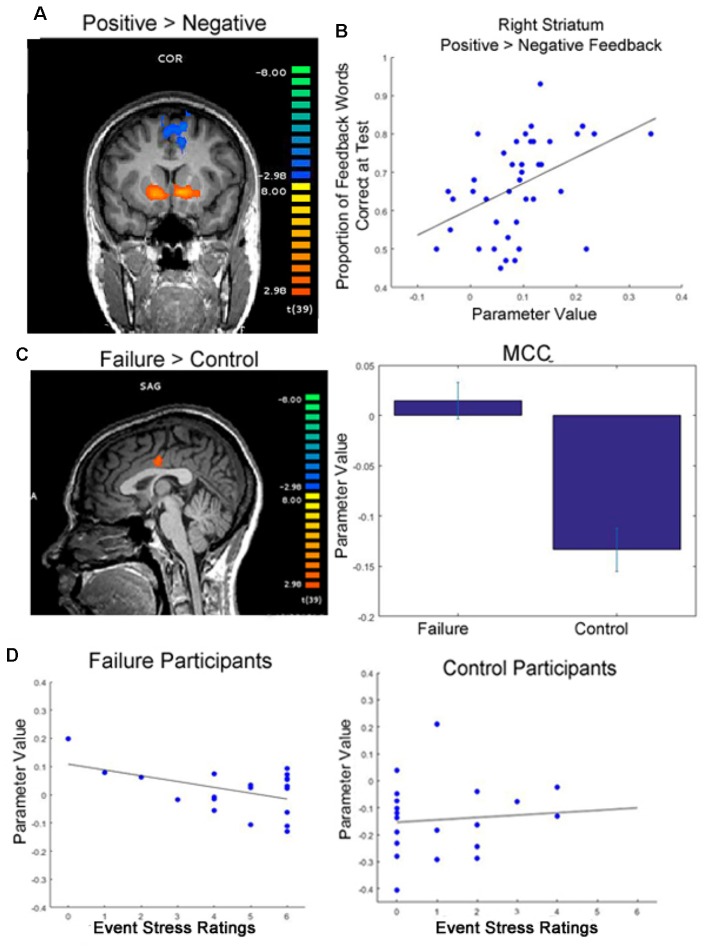
Functional magnetic resonance imaging (fMRI) results. **(A)** Participants showed greater activation in the striatum when viewing positive feedback than negative feedback during round 2 of the learning phase, when feedback was meaningful for performance (*p* < 0.05, cluster corrected; initial cluster forming threshold *p* < 0.001). **(B)** Greater differential activation in the right dorsal striatum when viewing feedback was significantly correlated with later memory for feedback words. **(C)** Participants who were randomly assigned to write about past failures showed greater activation in the mid cingulate cortex (MCC) while viewing target words throughout the learning phase than participants who wrote about a trivial topic (*p* < 0.05, cluster corrected, initial cluster forming threshold *p* < 0.005; peak activation coordinates in Talaraich space: −4, −11, 30). Whereas control subjects showed typical decreases in activation in the MCC during the task, failure subjects’ activation was near baseline averaged around 0 (right). **(D)** Individual differences in self-reported event stress ratings significantly predicted MCC deactivation for failure participants (left). We did not see this same relationship for control subjects (right).

For each participant, we modeled the 4-s word presentation screen (Slide 2 in Figure 1) and the 1-s feedback presentation screen (Slide 4) as regressors in our model. The regressors were convolved with a canonical hemodynamic response function. A predictor for missed trials (i.e., when subjects failed to respond on Slide 2 within the 4-s response window) was included in the model as a predictor of no interest. Additionally, the six motion parameters were also included in the model as predictors of no interest. For all analyses, we utilized the continuity-based cluster-level threshold estimator in BrainVoyager, with an initial significance threshold of *p* < 0.005. We then selected to run 1,000 Monte Carlo simulations, and corrected each contrast to a contiguity threshold cluster-level false-positive alpha rate of 0.05. Due to concern that this two-step cluster thresholding procedure is susceptible to inflated type 1 error (Eklund et al., 2016), we supplemented this analysis with permutation-based non-parametric tests, submitting images for each participant (contrast of beta weights from subject-level GLM estimation: positive feedback minus negative feedback; non-feedback trial word presentation beta weights) using the FSL randomize procedure with threshold-free cluster enhancement, using 10,000 iterations (Smith and Nichols, 2009). The non-parametric contrasting procedure provides further information on results that survive the more rigorous threshold. In regions of interest that survive this rigorous threshold (striatum and mid-cingulate cortex, MCC), peaks from the parametric analysis are identified for visualization (Figure 3) and further analysis (individual differences correlations and psychophysiological interaction (PPI) analysis).

#### Analysis of Feedback Across All Participants

We conducted a whole-brain analysis that examined activation at the time of feedback presentation during round 2 of the learning phase (i.e., when word matches are no longer arbitrary, therefore making feedback meaningful to choice). A contrast of interest included Positive Feedback > Negative Feedback to replicate previous results showing that this task elicits positive vs. negative feedback differences in brain areas typically associated with feedback processing (e.g., DePasque Swanson and Tricomi, 2014; Lempert and Tricomi, 2016).

#### Analysis Across Writing Groups

We also conducted a whole-brain analysis that examined group differences in activation between both writing groups (failure writing topic vs. control writing topic). Contrasts of interests included a contrast that examined activation differences in the failure writing vs. control writing groups at time of word presentation during the learning phase of the task, as well as contrasts that examined group differences (failure vs. control) in feedback processing (i.e., positive feedback overall, negative feedback overall, and positive vs. negative feedback).

#### Functional Connectivity Analysis

Additionally, we conducted a PPI analysis. Based on the results from our GLM analysis, we examined task-based functional connectivity between the MCC (signal time series from peak of the failure writing vs. control word presentation contrast) and other brain regions, for the failure writing group and the control group. We used the time period of the word presentation in the learning phase as the psychological context, because this is the time period in which we observed an effect in the MCC in our GLM analysis.

### Behavioral Analyses

As a manipulation check, an independent rater rated each of the writing samples on a 7-point Likert scale for negativity, emotional content, intensity, persistence, finding a “silver lining,” and relation to self. We expected the writing samples to vary greatly in content but expected the samples from the failure condition to be higher on each of these measures than the samples from the control condition.

We conducted *t*-tests that examined group-level differences in performance on the word association learning task. We looked at percent correct during round 2 of the learning phase (i.e., when choice is no longer arbitrary) excluding trials with no response, as well as performance differences within each feedback context (feedback condition vs. no feedback condition). We also repeated these tests for test phase performance. We also examined whether there were any significant correlations between our survey measures, performance, and brain activation in areas associated with writing group differences. Brain activation estimates were extracted from peak voxels in left caudate (positive vs. negative feedback contrast, all participants) and MCC (failure writing vs. control word presentation contrast). All analyses based on survey measures were exploratory.

## Results

### Behavioral Results

We found that there was a significant difference between groups in event stress ratings, with participants in the failure group rating the event they wrote about as more stressful than participants in the control group (failure writing group mean = 4.55, SD = 1.8; control group mean = 1.10, SD = 137; *t* = 6.77, *p* < 0.001). Additionally, the writing samples from the failure content were rated as more emotional (*p* = 0.02), more intense (*p* < 0.001), more related to the self (*p* < 0.001), and marginally (although not significantly) more negative (*p* = 0.071).

We also examined performance at each phase of the task. All analyses were performed after discarding missed trials (i.e., trials where participants did not respond within the 4 s response window). There was no difference in the number of missed trials between groups (failure writing group mean = 4.85, SD = 10.01; control group mean = 2.30, SD = 6.97; *t* = 0.94, *p* = 0.356). As expected, participants were at chance for round 1, when word matches were arbitrary and there was no way of knowing in advance which word would be the correct match, and there was no difference between groups (failure writing group mean = 52.63%, SD = 7.90%; control writing group mean = 53.81%, SD = 10.19%; *t* = 0.37, *p* = 0.7152). However, contrary to our hypothesis and the results of our behavioral pilot study (see Supplementary Figure S2), we did not find a significant difference in performance scores on round 2 of learning phase between writing groups, when performance depended on memory of the correct pairs from round 1 (failure writing = 58.28%, SD = 7.77%, control writing = 57.51%, SD = 9.70%; *t*_(38)_ = 0.279, *p* = 0.782), nor did we see differences when examining only words from the feedback (failure writing = 60.24%, SD = 8.64% control writing = 58.14%, SD = 11.61%; *t*_(38)_ = 0.65, *p* = 0.520) or no feedback rounds (failure writing = 56.32%, SD = 09.22% control writing = 56.87%, 10.11%; *t*_(38)_ = −0.18, *p* = 0.856). Furthermore, we did not see a significant difference between writing groups’ scores at test (failure writing = 61.90%, SD = 8.87%, control writing = 64.65%, SD = 9.55%; *t*_(38)_ = −0.94, *p* = 0.351), nor did we see significant differences between groups’ later memory for words learned in a feedback (failure writing = 65.90%, SD = 11.93%, control writing = 67.15, SD = 13.08%; *t*_(38)_ = −0.32, *p* = 0.754) or no feedback context (failure writing = 57.90, SD = 8.59%, control writing = 62.50, SD = 9.92%; *t*_(38)_ = −1.45, *p* = 0.156).

Overall, the average confidence ratings were not significantly different across groups (failure writing group mean = 5.41, SD = 0.88; control writing group mean = 4.93, SD = 1.21, *t* = 1.44, *p* = 0.157). However, across both groups, we found that confidence ratings at test significantly correlated with scores during round 2 of the learning phase (*r* = 0.43, *p* = 0.006), as well as greater later memory for words learned in a feedback context (*r* = 0.31, *p* = 0.05), but not the no feedback context (*r* = 0.05, *p* = 0.77).

Moreover, when examining correlations between behavioral results and survey measures, we found that self-reported scores on the Need for Cognition (NFC) scale significantly positively correlated with overall performance scores in round 2 (*r* = 0.32, *p* = 0.04), as well as for later memory of words learned in a no feedback context (*r* = 0.34, *p* = 0.035). We also found that self-reported desire to do well on the task, as well as task enjoyment, significantly correlated with performance during round 2 of the learning phase (care ratings, *r* = 0.33, *p* = 0.039; enjoy ratings, *r* = 0.32, *p* = 0.045). Last, age was significantly positively correlated with overall performance during round 2 of the learning phase (*r* = 0.573, *p* < 0.001), as well as test phase memory (*r* = 0.50, *p* = 0.001), task enjoyment (*r* = 0.33, *p* = 0.036), and scores on the NFC scale (*r* = 0.38, *p* = 0.016).

### fMRI Results

#### Across All Participants

Replicating previous findings (DePasque Swanson and Tricomi, 2014; Lempert and Tricomi, 2016), participants exhibited significantly more activation in the striatum (caudate and nucleus accumbens) for positive vs. negative feedback during feedback blocks in round 2 of the learning phase; see Figure 2 for whole-brain differences, and Table 1 for full brain results.

**Table 1:** Brain regions identified by GLM analysis.

Region	BA	Number of voxels (3 × 3 × 3 mm^3^)	Peak (Talaraich: x, y, z)	*t*
Feedback Presentation During Round 2 of Learning Phase (all subjects, *p* < 0.001, corrected to *p* < 0.05)				
*Positive > Negative*				
Right occipital gyrus	19	7,444	11, −102, −6	5.89
*Right putamen		1,732	14, 10, −6	6.02
*Left caudate head		1,720	−7, 10, −3	6.47
*Left occipital lobe	17	988	−16, −92, 12	5.14
*Negative > Positive*				
Superior frontal gyrus	6	2,058	−10, 1, 57	7.30
Thalamus				
		895	−13, −17, 6	5.61
Word Presentation During Learning Phase (across subjects, *p* < 0.005, corrected to *p* < 0.05)				
*Failure > Control*				
*Mid-cingulate cortex	23	531	−4, −11, 30	3.98
Left cerebellum		326	−31, −77, −30	3.64

*Indicates that the given peak survives the corresponding non-parametric permutation-based statistical contrast (corrected *p* < 0.05, full results in Supplementary Table S2).

To examine whether individual differences in brain activation correlated with performance measures, we examined whether individual differences in the strength of the positive vs. negative feedback contrast in this striatal region correlated with performance across all participants. We found a significant correlation between differential activation in the right striatum when viewing positive feedback contrasted with negative feedback during the task and later memory for words learned in a feedback setting (*r* = 0.35, *p* = 0.027). Thus, more differentiated activation in the striatum in response to feedback during learning resulted in better later memory for words originally learned in a feedback setting.

#### Across Writing Groups

While we did not see significant differences in feedback processing across groups, at the time of word presentation failure writing participants exhibited significantly greater activation in the MCC than participants who wrote about a trivial topic (Table 1 and Supplementary Table S2). Specifically, failure writing participants exhibited significantly greater activation in the MCC than participants who wrote about a trivial topic. To determine the direction of this relationship—for example, if failure participants exhibited greater activation vs. less deactivation than control participants, we examined the beta weights of the GLM of this contrast. After examining these parameter values, it became evident that while control participants exhibited decreases in activation in the MCC, the mean activation in the MCC for failure participants increased slightly from baseline; see Figure 3C for visualization.

Additionally, we conducted a PPI analysis that examined functional connectivity between the MCC and other brain regions. We found significant functional connectivity between the MCC and both the caudate and the medial prefrontal cortex (mPFC) in failure subjects at the time of word presentation. We further interrogated the role of the caudate and mPFC regions on behavior in our failure subjects but found no significant relationship between connectivity in these regions and performance on round 2 of the learning phase. We also conducted a second PPI analysis that examined MCC functional connectivity in control participants using the same procedures. We did not find that MCC activation significantly correlated with other brain regions in control participants. This finding was expected given that the MCC was more active in failure participants than control participants. These analyses are included in Supplementary Figure S1, Supplementary Table S1.

Because the MCC is typically deactivated during task engagement (Harrison et al., 2011), while increases in activation are typically associated with processing of negative emotion (Maddock et al., 2003; Shackman et al., 2011), we also examined how individual differences in MCC activation correlated with aspects of participants’ writing. Specifically, we found that within the group of participants assigned to write about a past failure, writing about more severe failures (self-reported by the participant) predicted greater deactivation in the MCC (*r* = −0.47, *p* = 0.038). We did not see this same relationship for control participants (*r* = 0.10, *p* = 0.680); see Figure 3D for an illustration of these correlations.

Additionally, we tested whether the ratings of the negativity of the writing samples were correlated with MCC activation for each of the groups. The correlation was not significant for either the control group (*r* = 0.32, *p* = 0.191) or the failure group (*r* = −0.074, *p* = 0.756), suggesting that heterogeneity in valence of the movie content or failure experience was not driving neural activation in the MCC.

## Discussion

Writing about a negative experience like a past failure has been shown to lead to a variety of benefits (Harber and Pennebaker, 1992; Pennebaker, 1997; Ramirez and Beilock, 2011; DiMenichi and Richmond, 2015). However, little is known about how writing and thinking deeply about a past failure could affect processing in the brain. Information about how expressive writing affects neural processing could offer valuable insight as to why previous studies have found that expressive writing leads to cognitive and emotional benefits.

When examining brain activation in all participants in our task, we found greater activation in the striatum when participants viewed positive feedback on the task as compared to negative feedback. This finding replicated previous work that suggests the striatum plays an important role in feedback processing (Tricomi and Fiez, 2012; DePasque Swanson and Tricomi, 2014; Lempert and Tricomi, 2016). Moreover, greater differences in activation in the striatum in response to positive vs. negative feedback resulted in better subsequent memory for words originally learned in a feedback setting. Our findings support previous research that has suggested that individuals who exhibit greater striatal sensitivity exhibit better error correction (Klein et al., 2007; Krugel et al., 2009; Ullsperger et al., 2014). When individuals are less affected by feedback (as evidenced by less differential response in the striatum) they may not learn as much from this type of feedback.

Furthermore, when examining differences across our two writing groups, we found that participants who were assigned to write about a difficult time in which they did not succeed exhibited greater activation in the MCC as compared to control subjects, who on average displayed decreases in activation from baseline. Our finding within control subjects may have represented typical deactivation of the MCC that is found when an individual is processing a task and therefore not processing information with an emotional context (Harrison et al., 2011). Asking an individual to reflect on a particularly emotional time in his or her life may have elicited increases in brain processing in the cingulate cortex, which has been implicated in processing negative emotion (Maddock et al., 2003; Shackman et al., 2011). Whereas the amygdala, which did not show differential activation between groups in our study, is heavily involved in processing negative emotions pertaining to vigilance, such as fear (Hamann et al., 2002), the cingulate cortex tends to be more involved in processing negative emotions that relate to the self, such as during one’s own experience of negative affect (Shackman et al., 2011). Moreover, the anterior region of the cingulate cortex (ACC) has been implicated in processing error detection, while the MCC is reported to play a vital part in processing information regarding negative emotion (Maddock et al., 2003). This region also tends to be more active while an individual experiences physical pain (Shackman et al., 2011). Therefore, participants who wrote about past failures may have shown greater activation in the MCC because they were recently asked to process highly emotional (and likely negative) information about their past failings. Future research is necessary to confirm that writing about failures truly induces negative emotion (e.g., by asking participants to rate their emotion after writing, rather than their stress level), and examine how these ratings relate to MCC processing. Paradoxically, when examining individual differences in failure writing and activation in the MCC, we found that self-reported event stress ratings actually predicted greater *de*activation of the MCC. Disclosure literature suggests that expressive writing about more intense negatives from one’s past may actually result in greater health, physical, and cognitive benefits (Harber and Pennebaker, 1992; Pennebaker, 1997). Furthermore, in longitudinal mindfulness interventions, individuals are trained to draw awareness to one’s thoughts and feelings in the present moment, and then slowly let go of negative or nagging feelings to focus on the current moment (Kabat-Zinn, 2009). Neuroimaging studies suggest that mindfulness training can result in reduced activation in the MCC during emotional stimuli (Farb et al., 2010). In the same way that drawing one’s awareness to negative emotions may result in greater deactivation of the MCC, writing about a past failure may also utilize similar neural processing in order to result in improved cognitive processes. Moreover, while writing about failures superficially may have resulted in increased emotional processing, reflecting on a particularly intense failure may have resulted in neural processing that more closely resembles not being exposed to emotional stimuli—i.e., the MCC deactivation exhibited by control writing participants. One possibility is that writing about more intense failures may allow an individual to better process negative thoughts before moving on to a new task.

Much of the work on the benefits of expressive writing has focused on how writing about very negative experiences, such as trauma, provides emotional benefits (Pennebaker et al., 1988; Harber and Pennebaker, 1992; Lepore and Smyth, 2002; Smyth et al., 2008). However, there is also evidence that writing about more universally experienced negative events and emotions, such as test anxiety and failure, confers benefits as well (Klein and Boals, 2001; Ramirez and Beilock, 2011; DiMenichi and Richmond, 2015; DiMenichi et al., 2018). Our neural results show that expressive writing about commonplace negative events, such as failure, leads to differences in neural processes during cognitive tasks similar to those encountered in school environments, such as memory tasks. This suggests that expressive writing has downstream effects not only for those who have endured very negative experiences, such as trauma but for almost anyone. This has implications for educational environments, as it shows how experiences that may seem unrelated to the task at hand, such as experiencing and writing about failure, can then influence neural processing during learning. Furthermore, since failure can be experienced within academic environments (e.g., failing a test or a class), strategies for overcoming these failures are particularly important. Indeed, previous work showing that writing about test anxiety helps decrease that anxiety and boosts performance suggests that expressive writing may be an effective tool for educators to use to address negative emotions stemming from classroom experiences.

Nevertheless, our fMRI results suggest that writing about particularly stressful failures may have led to MCC activity more like control participants, which is in line with previous findings that the benefits of expressive writing may be strongest when writing about strongly negative events (Harber and Pennebaker, 1992; Pennebaker, 1997). This may be one possible reason why, contrary to the results of our pilot behavioral study, we did not see significant performance differences across writing groups on our task. It is possible that behavioral differences between groups would be stronger if the writing topic was more strongly negative, such as a trauma. It is interesting to note that previous research has suggested that the beneficial outcomes of expressive writing may be related to positive aspects of writing, such as meaning making and affect labeling (Pennebaker and Chung, 2011; Memarian et al., 2017). In our dataset, externally coded scores based on the writing samples were not associated with behavioral or neural outcomes, whether they were based on the negativity of the writing samples or more positive aspects, such as persistence and finding a “silver lining.” Instead, it was the participants’ own ratings of the stressfulness of the event which correlated with MCC activation, lending support to the idea that the experience that the participant writes about may be at least as important as the content of the writing sample.

Although we did not observe behavioral differences between groups in our sample, differences in neural activation between groups in the absence of behavioral differences can still reveal important differences in cognition underlying behavior (Gilman et al., 2015). Behavioral results were also highly correlated with age in our sample, perhaps suggesting that younger participants had greater difficulty focusing on our learning task. Indeed, a recent study suggested that adults perform slightly better on this task than adolescents (DePasque and Galván, 2019). Furthermore, although we did not find a significant relationship between age and self-reported event stress ratings, perhaps younger participants were less likely to have experienced the *type* of failures that result in learning benefits after writing about them, especially considering that persistence improves as one ages, likely as a result of experience (Duckworth et al., 2007). Future studies might consider implementing our task on a sample with a slightly older mean age.

We conducted a PPI analysis that found that, among participants who wrote about past failures, the MCC may participate in a network of activation in conjunction with the caudate and mPFC. This activation may underlie differences in affective experience of the task. However, individual differences in activation in these regions did not predict any measures of behavior. Future directions may include measuring affect throughout the task in order to better parse the relationship between MCC and subsequent neural activation.

A potential limitation of our experimental design is that the content of the writing in our control condition was free to vary along many dimensions, including valence, based on the movie each participant chose to write about. However, ratings of negativity of the writing samples within the control condition were not significantly correlated with MCC activation, suggesting that heterogeneity in the negativity of the writing content in this condition was not driving activation in this region. Furthermore, the control condition was designed to be rich in episodic content, rather than completely neutral in valence, since writing about something fully neutral could have introduced a potential confound of boredom. Additionally, the study’s goal was not to investigate neural responses to the writing itself, but rather, how neural responses in a well-studied learning task differ based on the previous writing experience. That a 10-min writing exercise resulted in group differences in subsequent neural activation during an unrelated learning task emphasizes the underappreciated role of state-based differences in neural activation to the task at hand, which may be related to recent experiences, such as recalling and writing about a failure.

Broadly speaking, our findings suggest that writing about a past failure, especially a failure that one found to be particularly stressful, may be related to altered neural processing in the MCC. In addition to adding to our understanding of the mechanisms by which expressive writing influences cognition, our results have implications for educators hoping to improve learning, especially after students experience academic failure.

## Ethics Statement

This study was carried out in accordance with the recommendations of the Rutgers Institutional Review Board with written informed consent from all subjects. All subjects gave written informed consent in accordance with the Declaration of Helsinki. The protocol was approved by the Rutgers Institutional Review Board.

## Author Contributions

BD and ET designed the experiment. BD ran subjects through the task and performed statistical analyses. All authors contributed to advising on statistical analyses and writing the manuscript.

## Conflict of Interest Statement

The authors declare that the research was conducted in the absence of any commercial or financial relationships that could be construed as a potential conflict of interest.
