# Supplementary material for: Effects of Expressive Writing on Neural Processing During Learning
Source: Front Hum Neurosci. 2019 Nov 6;13:389. doi: 10.3389/fnhum.2019.00389 (PMC6869814; doi:10.3389/fnhum.2019.00389)
Supplement: Supplementary file 1 [file Data_Sheet_1.PDF]

Supplementary Material

PPI Analysis

We conducted a psychophysiological interaction (PPI) analysis that examined functional connectivity between the MCC and other brain regions at the time failure participants were viewing word matches (i.e., the cue screen).

MCC time series were extracted as mean signal in a 6mm radius sphere centered at the MCC group level contrast peak coordinate (-4 , -11, 30). We then added this time series to the first level model, along with psychophysiological interaction of this time series with the HRF-convolved regressor for word presentation events, and examined the group level contrast of this interaction regressor versus null in the failure writing group. At the statistical threshold of $p < 0.005$ corrected to $p = 0.05$, significantly activated regions included the left caudate, and bilateral medial prefrontal cortex (mPFC). The caudate is a region typically associated with reward processing during learning (Tricomi, Delgado, McCandliss, McClelland, & Fiez, 2006), while the mPFC has been implicated in both processing value (Plassmann, O'Doherty, & Rangel, 2010) and self-referencing (Amodio & Frith, 2006). See Supplementary Figure 1 and Supplementary Table 1 for the full activation table.

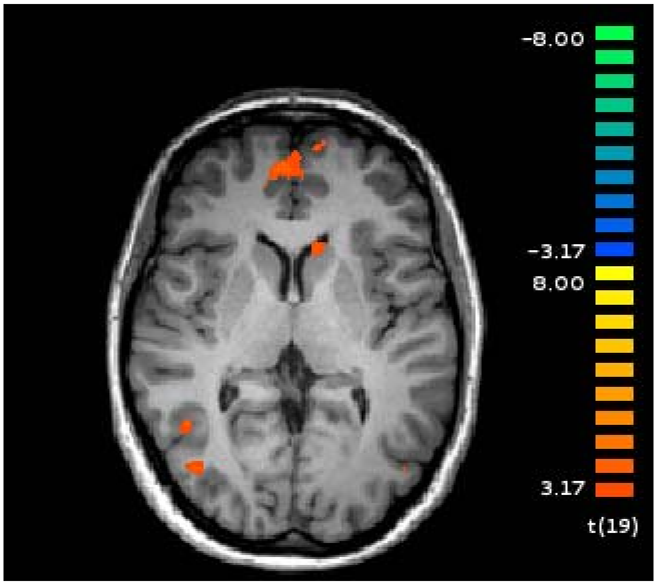

Supplementary Figure 1. In our PPI analysis, we found that MCC activation during word learning was significantly correlated with activation in both the medial prefrontal cortex, as well as activation in the caudate.

Supplementary Table 1.

Region	BA	Number of voxels (3 x 3 x 3 mm^3)	Peak (Talaraich: x, y, z)	*t*
Right superior temporal gyrus	39	1399	47, -53, 27	5.38
Right supramarginal gyrus	40	323	47, -41, 33	4.51
Right middle Occipital gyrus	19	441	38, -74, 3	5.65
Right superior frontal gyrus	10	153	41, 49, 21	4.67
Right medial frontal gyrus	11	169	23, 40, -3	4.09
Bilateral medial prefrontal cortex	10	2228	-4, 49, 0	5.16
Right anterior cingulate cortex	24	277	2, 28, -6	4.77
Left caudate		197	-10, 19, 6	5.12
Left occipital lobe	18	471	-16, -80, -6	4.59
Left middle frontal gyrus	11	593	-25, 43, -9	5.11
Left inferior temporal gyrus		308	-46, -71, 3	4.70

We also conducted a second PPI analysis that examined MCC functional connectivity in control participants using the same procedures. We did not find that MCC activation significantly correlated with other brain regions in control participants. This finding was expected given that the MCC was more active in failure participants than control participants.

Although we found significant individual differences moderators between the MCC and behavioral performance (see main text), we report no significant relationships between activity in the caudate and mPFC regions with performance. Furthermore, individual differences in caudate activation did not serve as a significant moderator/mediator in our mediation analysis.

Our results suggest that writing about a past failure may lead to increases in activation in the mid-cingulate cortex, and that both the caudate and mPFC may be involved in learning after processing the negative emotions associated with writing about a past failure. However, because these measures do not correlate significantly with behavior, we suggest interpreting these results with caution.

Results of Whole Brain Permutation Test of Primary Contrasts

Two primary findings in this manuscript are based on a two-step (1. cluster defining threshold, 2. $p<.05$ correction based on cluster size) cluster thresholding procedure: (a) a cluster of MCC activity exhibited greater signal during word presentation in failure writing participants compared to control writing participants, and (b) striatum signal was greater in response to positive compared to negative feedback. This procedure is known to be susceptible to an inflated type 1 error rate, and nonparametric permutation-based statistical thresholding is recommended to maintain type 1 error at the specified level ($p<.05$) without increasing type 2 error (Eklund, Nichols, & Knutsson, 2016). For this reason, we conducted a nonparametric permutation-based

test of the two contrasts, submitting images for each participant (contrast of beta weights from subject-level glm estimation: positive feedback minus negative feedback; non-feedback trial word presentation beta weights) using FSL's randomise procedure with threshold free cluster enhancement, using 10,000 iterations (Smith & Nichols, 2009). The results were thresholded at corrected p<.05 (two-tailed), and yielded substantively similar findings, including bilateral striatum for the positive versus negative feedback contrast, and similar MCC signal differences for the between participants comparison of failure writing versus control activity during word presentation. Supplementary table 2 gives the full results from this procedure.

Supplementary Table 2.

Region	BA	Number of voxels (3 x 3 x 3 mm^3)	Peak (Talaraich: x, y, z)	*t*
Feedback Presentation During Round 2 of Learning Phase (all subjects)				
Positive > Negative				
*Left Caudate		6365	-6, 11, -2	8.25
Right Caudate			15, 5, -8	7.28
Left Putamen			-21, 8, -8	6.68
Medial Prefrontal Cortex			-12, 44, -2	5.11
Right Occipital Cortex	17		12, -91, 7	5.04
Right Occipital Cortex	18		18, -82, 4	5.04
Right Occipital Cortex	37		39, -64, 1	5.03
Left Superior Temporal Cortex	41	25	-48, -37, 13	3.54
Negative > Positive				
(*No significant voxels)*				
Word Presentation During Learning Phase (across subjects)				
Failure > Control				
Mid-cingulate cortex	23	531	-3, -7, 37	4.51

*Contrasts thresholded at p<.05, corrected. Only clusters of more than 5 contiguous voxels shown. *Local maxima >12mm apart shown for clusters spanning multiple regions.*

Behavioral Pilot Study

We conducted a behavioral pilot study that followed the exact format of the main study (n = 25), only without fMRI, and that resulted in group differences during the word learning task (failure participants = 73.71%, SD = 9.37%; control participants = 63.54%, SD = 13.84%; t = 2.11, p = 0.047) and a difference that approached significance at test (failure participants = 68.92%, SD = 8.75%, control participants = 58.67%, SD = 15.10%; t = 1.96, p = 0.062)—see Supplementary Figure 2. The reason behind finding a significant difference during our pilot but

no significant behavioral differences across groups during the study featured in the manuscript remains unclear. However, importantly, we do observe significant neural differences across writing groups, which may help to explain previous effects of expressive writing on performance.

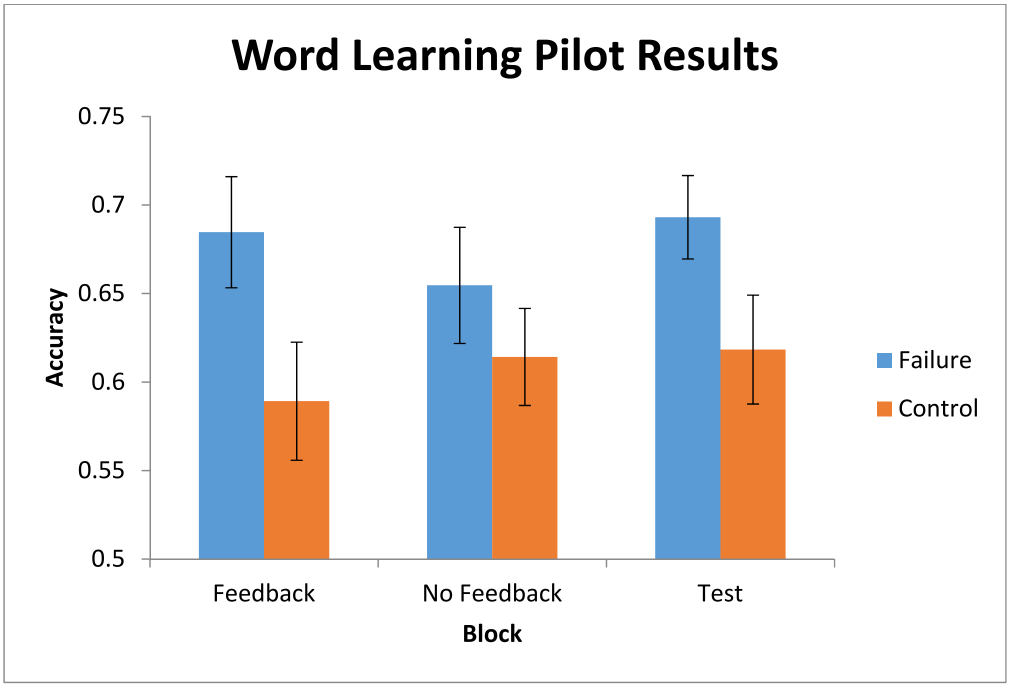

Supplementary Figure 2. Our behavioral pilot study revealed significant differences between writing groups during the task and marginally at test.

References

Amodio, D. M., & Frith, C. D. (2006). Meeting of minds: the medial frontal cortex and social cognition. *Nature Reviews Neuroscience*, *7*(4), 268-277.

Eklund, A., Nichols, T. E., & Knutsson, H. (2016). Cluster failure: Why fMRI inferences for spatial extent have inflated false-positive rates. *Proceedings of the national academy of sciences*, *113*(28), 7900-7905.

Plassmann, H., O'Doherty, J. P., & Rangel, A. (2010). Appetitive and aversive goal values are encoded in the medial orbitofrontal cortex at the time of decision making. *The Journal of Neuroscience, 30*(32), 10799-10808.

Smith, S. M., & Nichols, T. E. (2009). Threshold-free cluster enhancement: addressing problems of smoothing, threshold dependence and localisation in cluster inference. *Neuroimage*, *44*(1), 83-98.

Tricomi, E., Delgado, M. R., McCandliss, B. D., McClelland, J. L., & Fiez, J. A. (2006). Performance feedback drives caudate activation in a phonological learning task. *Journal of Cognitive Neuroscience, 18*(6), 1029-1043.
